# Evaluation of an online biomass probe to monitor cell growth and cell death

**DOI:** 10.1186/1753-6561-5-S8-P16

**Published:** 2011-11-22

**Authors:** Angelo Perani, Benjamin Gloria, Dongmao Wang, Anne-Sophie Buffier, Olivier Berteau, Geoffrey Esteban, Fiona E Smyth, Andrew M Scott

**Affiliations:** 1 Ludwig Institute for Cancer Research, Melbourne-Austin Branch, Heidelberg, Victoria, 3084, Australia; 2 Fogale Nanotech, Nimes, 30900, France

## Background

The estimation of cell density and cell viability of mammalian cell lines in cell culture has traditionally been performed using the exclusion dye trypan blue that stains “dead” cells when their cell membrane is damaged. In large scale cell cultures using bioreactors this estimation is performed off-line. The online biomass probe is based on the principle that under the influence of an electric field between two electrodes, ions in suspension migrate toward the electrodes. The cell plasma membrane is non-conductive so that the cells with intact plasma membranes are polarized and act as tiny capacitors and it has been shown that capacitance increases as the cell concentration does. The measurement is based on the linear relationship between the permittivity difference ε1- ε2 and the viable biomass concentration as it has been described by Ansorge et al. [1]. This study compares the data obtained using the biomass probe against the cell counts and viability determined by trypan blue exclusion with two GS-CHO cell line productions in bioreactors. Apoptosis determination measurements using rhodamine-123 and pan-caspase activation by flow cytometry will be compared to permittivity values.

## Material and methods

Cell Culture – Production: CHO-K1 SV cells were transfected with the vector coding for LICR C and LICR D antibodies using a Glutamine Synthetase expression vector (Lonza) and maintained in CD-CHO (Invitrogen) + 25mM of methionine sulfoximine (MSX) (Sigma). LICR C S1: 3L stirred-tank bioreactor (STR) (Applikon) in batch mode + temperature shift in CD-CHO + 32mM MSX (base 1); LICR C S2: 3L STR (Applikon) in batch mode + temperature shift in CD-CHO + 32mM MSX (base 2). LICR D S1: 3L STR (Applikon) in batch mode + 32mM MSX with addition of 4, 10 and 20 mM NH4+. Biomass: Online monitoring was performed with an iBiomass 465 system (Fogale Nanotech). Viable Cell Concentration – Cell size: Viable Cell Concentration and cell size were estimated by off-line measurements using an automated trypan blue cell counter (Cedex, Roche). Apoptosis: Apoptosis related measurements were performed with a FACS Aria III (Becton Dickinson) using rhodamine-123 (Sigma) at 1mg/mL in buffered saline (PBS) for 10^6^cells or with 10 mM of CaspACE™ FITC-VAD-FMK (Promega) for 10^6^ cells. FCS data was analysed with Gatelogic v3.08 (Inivai). Off-line measurement vs. on-line measurement: Correlation off-line and on-line measurements obtained by comparing the values of Xv.Dm4 to those of Δε (Xv: viable cell concentration estimated by Cedex in cells/mL; Dm: average diameter of cells estimated by Cedex in mm and Δε: cell permittivity measured by Fogale Biomass probe in pF/cm). Fluorescent Microscopy: 40μL of rhodamine 123 and CaspACE™ FITC-VAD-FMK stained samples were observed under fluorescent microscopy (FITC filter) with a 20x objective.

## Results

Permittivity vs. Cell Concentration: The comparison of the viable cell concentrations (Xv) profiles obtained with the Cedex and the permittivity profiles obtained with the biomass probe for the production of LICR C in bioreactors with different process controls show a clear correlation between the two measurements. The fitting of a linear regression curve gives a value of R^2^ = 0.8777. (Data not shown)

Diameter vs. permittivity related parameter: There is a direct correlation between the cell size (diameter) and viable cell concentration (Xv) measured by the Cedex and the permittivity measured with the biomass probe for the measurements obtained from LICR C bioreactor productions. The fitting of a linear regression curve gives a value of R^2^ = 0.8798. (Data not shown)

**Figure 1:**
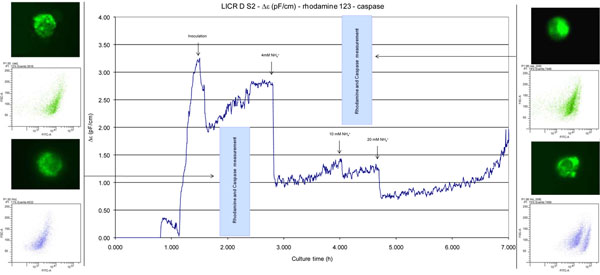
Detection of apoptosis using a pan- caspase inhibitor labeled with FITC and rhodamine 123 during early stages of cell culture using flow cytometry in parallel with the online measurement of Δε with the online biomass probe.

Apoptosis and permittivity: FACS analysis of a bioreactor run of LICR D cell lines under metabolic stress shows a different rhodamine-123 sub-population distribution (forward scatter (FSC) vs. rhodamine 123 channel) less than 2h after first stress signal. There is no change in the caspase distribution (FSC vs. caspase). These results were confirmed with visual observation of the cells by fluorescence microscopy (Figure 1) using the same samples that were maintained in a FACS-fixing buffer: 16g of D-glucose (Sigma), 40% formaldehyde solution (Sigma) in 500 ml of 0.01M (1X) PBS pH7.2, stored at +4 ^0^C.

## Conclusion

The measurements obtained with the biomass probe demonstrated correlation between viable cell concentration and permittivity. These observations suggest that it is possible to use such a probe in the routine monitoring strategy for production of biopharmaceuticals using GS-CHO cell lines in bioreactors. Early detection of apoptosis in bioreactor cultures seems possible by using measurements obtained with the biomass probe. This type of measurements can be of critical value when developing processes that aim to minimise apoptosis. Future experiments need to be performed to correlate these observations with process parameter changes and mitochondrial modifications.
